# Embodied Transformation: Psychedelic Therapy Through the Lens of Merleau-Ponty’s Phenomenology

**DOI:** 10.1007/s12124-026-10029-w

**Published:** 2026-09-21

**Authors:** Zinet Ritschel, Brady Wagoner

**Affiliations:** University of Copenhagen, Copenhagen, Denmark

**Keywords:** Psychedelic therapy, Mystical experience, Phenomenology, Embodiment, Merleau-Ponty

## Abstract

This paper develops a phenomenological account of the therapeutic significance of psychedelic-induced mystical experience, drawing on the philosophy of Maurice Merleau-Ponty. The central argument holds that the enduring therapeutic benefits of psychedelics emerge through a radical transformation in the structure of lived experience. Two prominent objections to the therapeutic value of mystical experience are examined and shown to rest on implicit Cartesian assumptions that obscure the embodied, situated character of human experience. Using Merleau-Ponty’s concepts of the body-subject and the intentional arc, depression is analysed as a collapse of perceptual attunement and engagement with the world, while psychedelic-induced mystical experience is shown to instantiate the opposite structure, recalibrating the intentional arc and restoring pre-reflective meaning. A phenomenological analysis of the acute mystical experience substantiates this claim, before a final response to the objection that psychedelic experiences are merely hallucinatory, arguing, via Merleau-Ponty’s account of perception as irreducible to passive reflection, that such experiences may still disclose meaningful dimensions of the world.

## Introduction

The past decade has seen renewed scientific interest in the therapeutic use of psychedelic substances, a shift often termed the psychedelic renaissance (Sessa, 2018; Hadar et al., 2023). Administered in controlled settings, psychedelics show significant potential in treating depression (Aday et al., 2020; Wheeler & Dyer, 2020; Andersen et al., 2021), with therapeutic outcomes correlating strongly with the occurrence of so-called mystical experiences: states marked by a sense of unity, sacredness and transcendence of ordinary space and time (Stace, 1960a; Ross et al., 2016; Ko et al., 2022). This effect extends even to healthy participants, among whom the intensity of such experiences predicts enduring psychological benefits (Nicholas et al., 2018). This correlation forms the central focus of the paper, which develops a phenomenological framework grounded in the philosophy of Merleau-Ponty to account for it, focusing on depression as a primary case among the many clinical applications of psychedelic research.

A psychedelic experience is an altered state of consciousness induced by substances such as psilocybin, LSD, or DMT (Letheby, 2021), commonly involving perceptual alterations, altered sense of time and space, intensified emotional states, and changes in self-experience, including ego dissolution, a temporary loss of self-boundaries (Nour et al., 2016; Haijen et al., 2018). Such experiences may also facilitate previously inaccessible thoughts or emotions, contributing to processes of insight and emotional release (Watts et al., 2017). In turn, a mystical experience is an altered state of consciousness, arising spontaneously or through deliberate induction, in which perception and self-experience undergo a qualitative shift beyond ordinary variation (Garcia-Romeu & Tart, 2013). Building on James’ *The Varieties of Religious Experience*, Stace (1960a, b) identified seven core phenomenal features of mystical experience: unity, transcendence of time and space, deeply felt positive emotion, a sense of the sacred, paradoxicality, ineffability, and a noetic quality. These features are operationalised via the psychometrically validated Mystical Experience Questionnaire (MEQ), based on Stace’s work (Barrett et al., 2015).

An important conceptual issue concerns the scope of the term ‘mystical experience’. The standard assessment treats mystical experience categorically, with instruments such as the MEQ30 classifying an experience as mystical above a threshold of 60%. Following William James’s (1902) foundational usage, however, this analysis treats mystical experience as a graded phenomenon rather than a discrete category, a view supported by Yaden et al.’s (2017) account of self-transcendent experiences as existing along a continuum from everyday states to rare, peak mystical episodes. This licenses a phenomenological account of mystical features as intensifications of structures already latent in ordinary perception, and extends the analysis to non-mystical psychedelic experiences, that is, those scoring below this threshold, that share this underlying structure. Some insights may generalise to non-psychedelic mystical experiences, but these fall outside the present scope. Henceforth, for brevity, ‘psychedelic experience’ and ‘mystical experience’ both refer to psychedelic-induced mystical states.

The paper proceeds as follows. It first examines two objections to the therapeutic significance of mystical experience, showing both to rest on implicit Cartesian assumptions, before introducing Merleau-Ponty’s concepts of the body-subject and the intentional arc as a framework for embodied, situated experience. This framework is then used to analyse depression as a collapse of the intentional arc, and to argue that psychedelic-induced mystical experience recalibrates this arc, restoring pre-reflective meaning and affective openness and thereby relieving depression, a claim substantiated through a phenomenological analysis of the acute mystical experience. The paper then addresses the objection that psychedelic experiences are hallucinatory, responding via Merleau-Ponty’s account of perception as never merely veridical, before considering implications for phenomenologically informed psychedelic research and concluding on how perception and embodied meaning are disrupted in depression and recalibrated through psychedelic-induced mystical experience.

## Psychedelic Therapy: From Cartesian Bias to Phenomenological Insight

Two key objections have been raised against the genuine therapeutic potential of psychedelic-induced mystical experiences. The first argues that such experiences are merely epiphenomenal, meaning that they are secondary to underlying neurological processes like increased neuroplasticity (Vollenweider & Kometer, 2010). Letheby (2021) challenges this on empirical grounds, noting that the available evidence suggests that mystical-type experiences, not merely neurological changes, are the strongest predictors of positive outcomes. The second objection, which Letheby (2021) terms the “comforting delusion objection”, suggests that the benefits arise from adopting emotionally reassuring but false metaphysical beliefs (e.g. a terminally ill person being comforted by adopting the belief that he will go to Heaven when he dies). However, Letheby refutes this by showing that such beliefs are neither reliably induced nor essential to therapeutic benefit.

In taking a closer look at the motivations for posing these objections, they appear rooted in an implicit Cartesian dualism, which separates mind and matter as distinct ontological substances (Baker & Morris, 2005). One reduces therapeutic effect to physical neural mechanisms; the other treats it as a disembodied act of propositional belief acquisition. Although explicitly dualistic models have largely been replaced by materialist paradigms in research, Cartesian thinking persists in models that either reduce mind to brain or detach it from the body (Matthews, 2004). Thomas Fuchs (2013) critiques this as reductive monism, replacing the soul with the brain while ignoring embodied unity of the person. Similarly, Mateo Sanchez Petrement (2023) highlights the Cartesian legacy of this split in what he terms “indivi/dualist” theories of self.

While the above objections aim to question the validity of the connection between mystical experience and therapeutic effect, they in fact reveal deeper conceptual commitments that shape how such experiences are interpreted. This is why they warrant attention: they illuminate not just resistance to the therapeutic efficacy of mystical experience, but the conceptual lens through which such efficacy is understood. If these objections can be traced to Cartesian presuppositions, then merely rebutting them may not suffice. Rather, these presuppositions must be transcended altogether. Overcoming this dualistic inheritance may require turning to phenomenology which explicitly rejects the Cartesian distinction between mind and matter and instead emphasises the lived, embodied nature of human consciousness taken in isolation from merely abstract theoretical structures of thought (Moran, 2002). In other words, the mind must be understood on its own terms.

In psychology, phenomenology constitutes a school of thought that is fundamentally anti-reductionist, rejecting the notion that mental illness can be reduced to maladaptive brain states (Langdridge, 2018). Instead, it seeks to illuminate the structures of meaning inherent in lived experience. Rather than simple introspection, this should be understood as a systematic endeavour to describe the fundamental structures underlying conscious experience and to explore how these structures might be disrupted in pathological conditions. While it begins with first-person descriptions, its aim is to uncover the underlying structures of consciousness, such as how perceptual meaning is formed or how temporal continuity is maintained. The emphasis lies on the organisation and architecture of experience, rather than its concrete content (Fuchs, 2002).

The relevance of a phenomenological approach to psychedelic therapy has not gone unnoticed. A growing body of phenomenological work aims to elucidate the nature of mystical experience through the lens of Matthew Ratcliffe’s (2008) theory of existential feelings (Letheby, 2021; Miceli McMillan & Jordens, 2022; Sanchez Petrement, 2023; Tijhuis et al., 2025). Within this framework, existential feelings are understood as pre-intentional, bodily orientations that structure conscious experience as an implicit relation between self and world, as well as being dynamically intertwined with personal narratives. The main mechanism of psychedelic therapy is understood as a result of an experientially induced shift in existential feelings.

Key phenomenological concepts that will be employed throughout the present paper include the body-subject (the lived experience of embodiment), and the intentional arc (the unified bodily-cognitive-affective directedness towards the world) drawn from the philosophy of Maurice Merleau-Ponty. This account does not seek to displace Ratcliffe’s (2008) theory of existential feelings, but to ground it explicitly within the dynamics of the body-subject and the intentional arc, extending this existing phenomenological work beyond a general account of existential change. The same concept of the intentional arc used to characterise the collapse distinctive of depression (Sect.  "Depression as Phenomenal Disconnection") is used to characterise its recalibration in mystical experience (Sects. "Psychedelic Experience as Phenomenological Restoration"  and "Returning to Mystical Experience"), yielding a unified rather than merely parallel explanatory structure. In the following section, the paper will elucidate these concepts, as they will be central to the subsequent analyses of both depressive and mystical states.

## Merleau-Ponty’s Phenomenology of Embodiment

### The Body-Subject: Embodiment and the Pre-Reflective Ground of Perception

Merleau-Ponty rejects two dominant Cartesian-inspired accounts of perception. On one hand empiricism reduces perception to passive sensory data, while on the other hand intellectualism overemphasises conscious thought and representation. Interestingly, these categories correspond closely to the criticism of the objections raised against mystical experience as therapeutic mediator discussed earlier. In contrast, Merleau-Ponty argues that perception is a pre-reflective, embodied engagement with the world. It is neither passive sensation nor detached cognition, but grounded in lived experience (Merleau-Ponty, 2013). To say that it is pre-reflective means that it exists prior to deliberate reflection or conceptual thought. For Merleau-Ponty, perception resists binary classification as either subjective or objective, internal or external, mental or mechanical. Thus, human beings are best understood as embodied subjectivities - a conception referred to in the literature as the body-subject (Wyllie, 2005). My body is not merely an object which my mind inhabits, but the body is “my point of view on the world” (Merleau-Ponty, 2013, p. 99).

Crucially, for Merleau-Ponty, perception is not passive reception of raw sensory facts. Instead, it is an active structuring of the phenomenal field, a term denoting the world as it appears in lived experience. He describes the phenomenal field as a “transcendental field” (p. 61). This means that the content of perception is not given as objective or neutral, but is shaped by “possibilities, impossibilities, and necessities” (Carman, 2019, p. 99). That is, what is perceived is shaped by the concrete ways in which the world is available or unavailable to the embodied subject, and these structures can vary between individuals and even for the same person across different contexts or times.

Hence, the phenomenal field is a field of possible actions (and not merely a collection of neutral objects) which becomes accessible to the body-subject in perception, and is molded by bodily ability and experience. In other words, the way objects are perceived is partly shaped by the body’s pre-reflective grasp of what actions are possible. For instance, an accomplished climber might perceive a rock-wall as *a way to the top of the rock*. An untrained person might perceive the same rock-wall merely as an obstacle. Hence, as Merleau-Ponty (2013) writes: “Consciousness is originally not an “I think that,” but rather an “I can.”” (p. 139). In this sense, the body-subject is not merely situated in the world but fundamentally intertwined with it in perception. In what follows, the discussion turns to Merleau-Ponty’s broader notion of the intentional arc, which elaborates this interrelation by demonstrating that the structure of the phenomenal field is not determined by physical capacities alone. Rather, it emerges through the dynamic integration of perception, habit, affect, and memory, all of which converge to shape how the world is meaningfully disclosed in experience. This provides a deeper understanding of how experiential meaning emerges at both simple and more complex levels.

### The Intentional Arc as the Structure of Experiential Meaning

To Merleau-Ponty, the body is not just biologically functional, but the original site of both simple and complex processes of meaning-making (Merleau-Ponty, 2013, pp. 147–148). Motor habits (whether learning to walk or playing an instrument) are not mechanical routines but ways the body takes up new modes of engaging with the world. Once a habit is acquired, it reflects a reorganisation of embodied directedness towards the world: a new structure of meaning sedimented in the body. Again, this meaning is pre-reflective - immediate and lived, rather than conceptual or intellectual. The body “understands” through its capacity for embodied engagement. Reflection may articulate meaning after the fact, but it does not generate it. As such, the world is not encountered as neutral and meaningless until interpreted; it is always already affectively and practically meaningful to the embodied subject.

To describe how such embodied meaning coalesces over time, Merleau-Ponty (2013) introduces the notion of the *intentional arc*. The intentional arc integrates past experiences, motor skills, emotional dispositions, and learned meanings into a cohesive orientation that guides both perception and action. It is through the intentional arc that the world appears as a field of both possible actions as well as values, and meanings. For instance, a skilled and enthusiastic guitarist does not perceive a guitar merely as a physical object, nor merely as something which *can* be played, but as something that *calls for* musical engagement. Similarly, a person marked by past trauma or cultural conditioning may perceive mice not as neutral creatures but as threatening presences which warrant fleeing from. As Merleau-Ponty (2013) writes: “[The] intentional arc creates the unity of the senses, the unity of the senses with intelligence, and the unity of sensitivity and motricity” (p. 137). He further notes that in pathological conditions, this arc may “go limp” (p. 137), hindering seamless or constructive engagement with the world.

Thus, through the intentional arc the world appears as inherently meaningful. The subject is not exposed to a neutral, objective world which must then be infused with deliberated, intellectual meaning. Rather, our embodied orientation towards the world imbues it with meaning. When I am afraid, I do not simply perceive myself as frightened, I perceive the world as frightening. In this sense, perception, affect, and meaning are holistically intertwined. Trauma, emotional disruption or even neurological damage can disturb this arc, diminishing the subject’s ability to engage constructively with the world - an issue that will be central to the subsequent analysis of depression.

To summarise: In the explication of Merleau-Ponty’s concepts, this section has presented a coherent phenomenological conception of subjectivity which entails that (1) perception is not simply passive reception of stimuli, but rather an active and bidirectional, co-constitutive engagement with the world, and that (2) qua the intentional arc, pre-reflective meaning is intrinsic to the phenomenal field. Thus “our body is not an object for an ‘I think’: it is a totality of *lived meanings* that moves toward its equilibrium” (p. 190, emphasis added). On the basis of this conception, the discussion will now proceed to present a phenomenological analysis of depression.

## Depression as Phenomenal Disconnection

### Depression as Disconnection: Fuchs’ Phenomenological Account

Depression is classified as an affective disorder, primarily involving mood disturbances linked to negative thoughts, self-evaluations, and emotions like anxiety, shame, and guilt (World Health Organization, 2019; American Psychiatric Association, 2013). Other common symptoms may include loss of appetite, weight, libido, insomnia, and psychomotor inhibition, but such somatic symptoms are considered secondary. The dominant psychological approach, cognitive therapy, views depression as distorted thinking and faulty information processing (Fuchs, 2013). Accordingly, cognitive behavioural therapy (CBT) is widely regarded as the treatment of choice. As such, in Western contexts, depression is predominantly conceptualised either as a mood and affective disorder or as a form of cognitive distortion.

However, Thomas Fuchs argues that cross-cultural psychiatric research reveals striking variations in how depression is experienced and expressed (Fuchs, 2005a, b, 2013). In many non-Western societies, depression manifests primarily through bodily or social complaints (Gureje et al., 1997). This suggests that the Western tendency to conceptualise depression as an internal mental disorder may reflect Cartesian biases: a privileging of mental content over embodied or relational suffering. Fuchs argues that depression is the same in all cultures, but that Western society lacks the non-dualistic vocabulary to adequately describe it. He goes on to offer a phenomenological perspective, presenting depression as what he calls a “detunement” of the lived body, namely a state where the individual experiences a disconnection from their environment and social world (Fuchs, 2005b, 2013). He emphasises that depression involves a breakdown in embodied and emotional attunement with others, and argues that depression is not simply something internal, but a profound disruption in one’s way of being in the world. As Fuchs puts it, “[T]he illness is not in the patient, but the patient is in the illness.” (2013, p. 222). In what follows, this account will expand on this phenomenological approach using the concepts of Merleau-Ponty.

### Merleau-Ponty on Trauma

Merleau-Ponty does not touch directly on the topic of depression. However, his account of traumatic experience as a disruption in the intentional arc, specifically as a stagnation in the inhabitation of particular worlds or perceptual structures, will lay the groundwork for the analysis of depression as a similar structural disruption. Given the established link between traumatic experiences and subsequent depression (Mandelli et al., 2015; O’Donnell et al., 2004), there is strong reason to believe that trauma contributes to depressive symptoms, thereby supporting this analysis. Merleau-Ponty (2013) writes that for a traumatised person:

“One present among all of them thus acquires an exceptional value. It displaces the others and relieves them of their value as authentic present moments. We remain the person who was once committed to this adolescent love, or the person who once lived within that parental universe. New perceptions replace previous ones, and even new emotions replace those that came before, but this renewal only has to do with the content of our experience and not with its structure. Impersonal time continues to flow, but personal time is arrested.” (p. 85).

Hence, the traumatised subject is locked into one particular experiential world (i.e. one kind of phenomenal field) at the expense of other possible such worlds. To illuminate this dynamic, John Russon (2003) offers the example of a child who, after suffering abuse from his brother during family dinners, develops a lasting fear of eating with others. Even as an adult in a new environment, he experiences intense anxiety during college dinners. As described in the above quote, for the traumatised person the present moment engages with the substance of experience (a dinner) but leaves its underlying structure unchanged (the dinner perceived as threatening in the absence of present threats). The traumatised man is unable to respond to the qualities of dinner with friends which would render it, in a potential world, pleasant. He *cannot perceive it as good*. Instead of shaping the present as a source of possibility, trauma causes the past to emerge as an obstacle.

### Depression as Collapse of the Intentional Arc

Building on Merleau-Ponty’s reflections on trauma, this section will articulate a phenomenological understanding of depression. Recall that the phenomenal field is transcendental, shaped by perceived “possibilities, impossibilities, and necessities” (Carman, 2019, p. 99). In this sense, one might claim that there is more than one possible present, given that the salience and valence of the same objects may present to consciousness in several ways. In trauma “[o]ne present among all of them […] acquires an exceptional value.” (Merleau-Ponty, 2013, p. 85). The same might be the case in depression, where a disruption of the body-subject’s intentional arc - a breakdown in the pre-reflective capacity to perceive and engage meaningfully with the world - may result in the phenomenal field losing its vitality and no longer calling for action or interaction; consequently, it appears flat, heavy, and inert, stripped of significance, no longer soliciting involvement. The guitar no longer calls for musical engagement; it appears as a (at best) neutral object. This interpretation finds strong resonance in first-person accounts of depression, in which individuals frequently describe a world emptied of value, where nothing beckons engagement and meaningful connection seems unreachable.

For example, in explaining the immobilising effects of his depression, a 48-year old man states that: “[W]hen you’re really depressed, you know, if you’re in your bedroom and someone said there’s a million dollars on the other side of the room and all you have to do is swing your feet over the edge of the bed, and walk over and get the million, you couldn’t get the million. I mean you literally couldn’t.” (Karp, 2017, p. 78).

This account reveals a disjunction between cognitive recognition and embodied orientation. The subject understands the situation conceptually - he knows that the imagined money is valuable and attainable - but is incapable of responding to it as a practical or affective possibility. In Merleau-Ponty’s terms, the intentional arc has collapsed. The million dollars, though intellectually acknowledged, no longer solicits action. The object does not appear within his phenomenal field as imbued with desirability or urgency. What to the healthy subject would constitute an obvious affordance for action is, to the depressed individual, experientially inert. The result is a profound stagnation.

A 31-year old woman explains how in a state of depression “You look at the world, the array of things that you could do, and they’re completely meaningless to you […] And you come to this terrible still point where there’s no reason to move because there’s nothing out there for you.” (Karp, 2017, p. 81). This supports the notion that the world is perceived as meaningless by the depressed subject. Again, the possibilities for actions are grasped intellectually, but the pre-reflective dimension of perception is absent, rendering them existentially meaningless.

A 37-year-old woman further underscores this phenomenological constriction: “[Now] I look at the world differently. Like I look at things sort of as gloomy and very precarious. The world is a very precarious place, and very hostile place, and so I tread very … carefully.” (Karp, 2017, p. 84). Here, the intentional arc is distorted in such a way that the world is encountered as threatening. This exemplifies Merleau-Ponty’s claim that perception is coloured by affective orientation - one does not merely feel depressed, but perceives the world as depressing. Finally, an anonymous patient highlights the disconnect between intellectual and pre-reflective engagement when they state that: “I would look at orchids and intellectually understand that there was beauty, but not experience it.” (Watts et al., 2017, p. 527). In this case, the perceptual object remains unchanged, yet its aesthetic and affective dimensions have become experientially inaccessible.

Interestingly, the state of depression can be understood as experientially mirroring the ideas of Cartesian dualism; the subject finds itself lodged in a body and in an environment from which it is fundamentally alienated. Petrement (2023), making the same observation, writes that: “Far from being the normal state of things, therefore, the Cartesian dream of closed individuality located in the mind is precisely descriptive of depression, and this points towards the therapeutic need to connect the self to something other than itself” (p. 38).

In sum, a phenomenological approach to depression, as developed by Thomas Fuchs and illuminated by Merleau-Ponty’s reflections on trauma, moves beyond biomedical and cognitive models that conceptualise depression as a purely internal dysfunction of mood or thought. Instead, it reveals depression as a profound collapse of the intentional arc that binds the self to a meaningful environment. The depressed individual does not merely think negatively or feel sad; they experience a breakdown in their very ability to be moved by, engage with, or find meaning in the world around them regardless of their intellectual reflections.

In the following section, this paper presents a phenomenological analysis of the therapeutic effects of psychedelic-induced mystical experience, with the aim of elucidating how such experiences may facilitate a re-attunement to the world.

## Psychedelic Experience as Phenomenological Restoration

### Petrement’s Theory of Immersive Reflection

Petrement (2023), who coined the term “indivi/dualist” to describe the common Cartesian-inspired view of the psychiatric subject as a separate, self-contained individual, offers a phenomenological perspective on psychedelic therapy. A crucial point of Petrement’s analysis is that the mechanisms of psychedelic therapy can be understood not merely as a transformation of the self (from depressed to not-depressed), but as a transformation of (lived) reality as such.

Petrement’s main argument for this view is that depression and psychedelic experiences instantiate opposing experiential *structures of possibility.* He argues firstly that the self is a field phenomenon - namely, that the self is partly constituted by an embodied orientation towards the world. Secondly, he argues that we (normally) experience reality as a dynamic possibility space. We orient ourselves in accordance with what we can *do*, and the boundaries of action determine how our orientations are structured. This structure is called a horizon which Petrement (2023) describes as “(…) a relational fact whereby one and the same environment *offers* different possibilities to different bodies, who in turn respond to or call forth different aspects of the former” (p. 40, italics in original). What distinguishes the experience of the depressed from the non-depressed subject is their respective horizons, or structures of possibility.

Petrement picks up Fuch’s (2013) notion of depression as bodily detunement as being an expression of patient inhabiting a disconnected world. Depression instantiates a sense of reality in which the subject is disconnected from the world and thus its possibilities of (inter)action; consequently, the subject inhabits a world which is bereft of possibilities. In contrast, the psychedelic experience connects the subject to the world in a radical manner, hence facilitating this sense of possibility. Petrement introduces the term *immersive reflection* to explain the process by which the therapeutic mechanisms of psychedelics operate. Unlike traditional forms of intellectual reflection, immersive reflection plunges the subject more deeply into embodied engagement. Under the influence of psychedelics, patients are drawn into intensified contact with bodily sensations, emotions, memories, and the environment. This immersive engagement facilitates the reorganisation of meaning structures, enabling patients to revisit traumatic experiences, rediscover forgotten capacities, and reorient themselves towards the future.

### Psychedelic Therapy and the Intentional Arc

Petrement’s understanding of the therapeutic effects of psychedelics is clearly compatible with Merleau-Ponty’s philosophy. The notion of the self as a field phenomenon corresponds to Merleau-Ponty’s idea that the body-subject’s existence is always co-constituted by its orientation towards the world. Petrement’s notion of reality as a possibility space aligns with Merleau-Ponty’s notion of the phenomenal field as transcendental and constituted by “possibilities, impossibilities, and necessities” (Carman, 2019, p. 99).

On this view, immersive reflection can be understood as a restoration of the intentional arc. While the intentional arc is never absent, it is disordered in depression. ‘Restoration’ thus refers not to the recovery of a lost structure, but to a reactivation of its integrative function, namely a renewed attunement to affect, memory, sensation, and world. This reattunement is not abstract or intellectual. Rather, it is a pre-reflective existential shift in how the world is lived, precisely the level at which Merleau-Ponty sees all human experience unfolding. This aligns with Petrement’s view that both depression and psychedelics alter the very structure of our lived experience by modulating how the self orients towards possibilities. One might object that immersive reflection sits awkwardly with Merleau-Ponty’s own emphasis on reflection as a distanced, deliberate act; however, for Merleau-Ponty reflection is never a view from nowhere but is itself performed by an embodied subject, so an intensified, embodied mode of access to lived experience is continuous with, rather than opposed to, his account of reflective method. While depression is a narrowing of possibilities resulting in a dead or alien world, the psychedelic experience facilitates a reorientation towards these possibilities - a perceptual recalibration. This interpretation finds support in first-person accounts attesting to the enduring perceptual shifts following psychedelic therapy.

One participant who was treated for depression describes how after their psychedelic experience: “Things look different even now. I would look over at the park and it would be so green, a type of green I’d never experienced before. Being among the trees was incredible, like experiencing them for the first time, so vibrant, so alive.” (Watts et al., 2017, p. 531). This demonstrates clearly a tangible difference in visual perception, signifying that the aesthetic qualities of the environment have acquired a new perceptual salience.

Other patients describe a recalibration of valence, where otherwise attractive but ultimately self-destructive things now appear in a less seductive light. For example: “I lost a lot of weight just purely because I didn’t want to eat badly and that went on for some months. I couldn’t eat what I knew wasn’t good for me. And I couldn’t watch pornography after the second dose. I started feeling it was sullying and not wholesome. I just didn’t want to do that.” (p. 532–533). Note that this aversion towards unhealthy food and pornography is not a matter of intellectual reflection or value judgement, but rather a shift in pre-reflective meaning. These objects no longer solicit desire because their perceived meaning within the phenomenal field has been fundamentally restructured. They are no longer perceived as truly desirable.

One patient states that: “Depression is like when you have a dead leg and the psilocybin made the bloodflow start to come back.” (p. 542). Another patient describes how the psychedelic experience instilled in him the feeling that: “The world is endless and I’m free to do whatever I want.” (McGovern et al., 2025, p. 20). Both statements support an understanding of the psychedelic experience as restoring an intentional arc which allows the subject to engage seamlessly and affectively with the world or, in Petrement’s terms, which expand their structure of possibility.

### The Enduring Effect

While Merleau-Ponty (2013) investigates cases of permanent neurological disruption to illuminate the structures of perception in both normal and pathological states, the psychedelic experience seems to present a distinct challenge. Its acute phenomenological alterations are transient, often lasting only a few hours. Yet, individuals frequently report sustained relief from depressive symptoms long after the immediate effects have subsided. This raises a critical issue: how can such fleeting perceptual transformations yield lasting therapeutic change? It seems intuitively plausible that the intentional arc might be affected under the acute influence of a drug, but what happens afterwards?

From Merleau-Ponty’s perspective, the answer lies not in the duration of the psychedelic state, but in the resulting reconfiguration of perceptual orientation. Psychedelic experiences initiate a re-structuring of one’s habitual body-world relation. This shift can sediment into new dispositions, perceptions, and affective openness. In this way, the intentional arc is not merely perturbed, but recalibrated, allowing for a renewed sense of agency and world-involvement long after the pharmacological effects have faded. This makes sense given the evidence that therapeutic effects are mediated by the resulting experience rather than being a direct effect of the drug itself (Letheby, 2021). To support this understanding, one might point to traumatic occurrences as constituting a mirror-image of this effect. As shown above, trauma constitutes a disruption of the intentional arc with lasting consequences. A violent assault, although brief in duration, might alter a person’s intentional arc in such a way as to result in anxiety persisting long past the event itself (Merleau-Ponty, 2013). As previously noted, trauma is strongly correlated with depression (Mandelli et al., 2015; O’Donnell et al., 2004). Hence, it is not unprecedented that singular and temporally limited experiences can have profound and enduring effects on the intentional arc. This experiential transformation may be likened to a *Gestalt shift*, which consists not in the acquisition of additional representations, but a reconfiguration of the pre-reflective organisation of experience. In the familiar duck–rabbit figure, the image remains identical; what shifts is the structure of the perceptual field. Crucially, once the second configuration is disclosed (say, from rabbit to duck), perception is irreversibly altered. One cannot simply “unsee” it, but the duck now remains continuously available to perception. In a similar sense, the psychedelic experience may recalibrate the intentional arc by disclosing an alternative organisation of self–world relations that remains sedimented as a possibility, even after the acute state has passed. It should be acknowledged that the notion of a gestalt shift functions here merely as a structural analogy. The general claim is more modest: phenomenology gives independent reason to expect that brief experiences can restructure the intentional arc; whether this expectation holds, and for whom, remains a question for the longitudinal empirical research.

Tijhuis et al. (2025) identify two requirements for psychedelic-induced mystical experiences to induce long-lasting effects on existential orientation. Firstly, the experience of oceanic boundlessness (i.e. dissolution of self-world boundaries) must be relatively strongly experienced. Secondly, the experienced ego dissolution which accompanies this experience must be positively valenced. Thus, not all psychedelic-induced mystical experiences *necessarily* have a therapeutic effect, but a desirable recalibration of the intentional arc requires the psychedelic experience to be sufficiently salient as well as (largely) positively felt. Finally, it is worth mentioning the importance of ‘integration’, namely the process of making sense of and incorporating insights from psychedelic experiences into daily life to support long-term therapeutic effect (Bathje et al., 2022). Integration practices can include journaling, therapy, rituals and nature-based activities, and can be understood as a continued effort to maintain the changes to the intentional arc prompted by psychedelics by sedimenting them into the intentional arc via repetition.

This section has examined how psychedelic experiences may recalibrate the subject’s intentional arc, thereby facilitating enduring therapeutic transformation. In this light, the mystical experience emerges as a structural inverse of the depressive condition. Where depression entails a collapse of perceptual openness and existential withdrawal, the mystical state appears to restore affective vitality and re-establish a meaningful relation between self and world. To further support this analysis, the discussion will now turn to a Merleau-Ponty inspired account of the phenomenal features characterising the acute mystical experience.

## Returning to Mystical Experience

Having outlined the perceptual shifts that may follow a psychedelic experience, this analysis will now examine some key phenomenal features of the acute mystical experience in greater detail, in order to further support the claim that such experiences involve a transformation of the intentional arc. This analysis will primarily draw on the central dimensions identified in the MEQ30: mystical unity, transcendence of time and space, and ineffability. However, in place of the MEQ30’s fourth “positive mood” dimension, the discussion will instead directly address the noetic quality (Stace, 1960a), which is considered more phenomenologically significant.

### Mystical Unity

At the heart of the mystical experience lies the loss of the usual sense of bounded, separate selfhood (Nour et al., 2016), often manifested as an experience of unity with the world. From a Cartesian-informed perspective on subjectivity, such experiences are deeply suspect, suggesting confusion between self and world. From a Merleau-Ponty inspired perspective, the self-transcendent quality of mystical experiences can be interpreted not as a breakdown of the self, but as a shift in the structure of self-world relation. When individuals under psychedelics report a felt loss of separateness or merging with nature, this does not signal confusion between internal and external reality. Rather, it reflects a softening of habitual ego boundaries, which ordinarily structure perception in terms of personal projects and defences. In such moments, the arc may expand to encompass a broader horizon of significance, where the distinction between self and world is reconfigured. This may explain the common reports of unity, peace, and deep interconnection in mystical experiences: the world is not passively observed, but affectively and perceptually lived through in a more porous, open way. In this light, ego dissolution is not the loss of self but the radical recovery of relational being. The therapeutic potential of such transformation is especially clear if one recalls that this openness may be precisely what is foreclosed in depression. As Petrement (2023) observes, in depression the Cartesian fantasy of isolated selfhood becomes an existential reality. The mystical experience, by contrast, may be understood as its exact structural inverse: a profound re-engagement with the world so radical that it temporarily dissolves the experiential distinction between subject and object.

### Ineffability

Another core quality of mystical experience is ineffability, namely the difficulty people report having with adequately communicating their experiences verbally. This may support the idea the psychedelic experience operates on a pre-reflective (and therefore pre-verbal) level. Pre-reflective experience is lived before being conceptualised or articulated; such experience occurs at a level prior to thought. This ineffability may correspond to the way that I might find it difficult to verbally articulate how I know that I need to stretch my arm and angle my hand *like this* in order to catch *that ball.* Nor can I necessarily justify why I find taking a walk a pleasant and desirable activity. I can talk of the tall green grass, the mild breeze, the blue sky, but I cannot *fully* articulate the appeal of these things to me. I just want to take a walk. I feel drawn to the activity in a way that resists explanation because it is first and foremost lived rather than (merely) thought. If psychedelic experience strongly attunes the subject to the pre-reflective engagement with the world, then naturally this particular kind of engagement resists full articulation. It is exactly this pre-reflective engagement which is deficient in the depressive state where the value of things may be intellectually grasped, but not experienced.

### Noetic Quality

A Merleau-Ponty inspired approach may further help clarify the mystical experience’s postulated noetic quality, namely the sense that the experience somehow signifies ultimate reality and that in undergoing it one has gained some kind of insight or knowledge. If, qua Merleau-Ponty, meaning-making is fundamentally pre-reflective and from an embodied, intuitive engagement with the world, then a shift towards this mode of perception may be perceived as a close communion with something deeply meaningful because the world is no longer scrutinised from a distance but lived through directly. As such, what is encountered may feel not only real but *more real than ordinary reality* precisely because it bypasses intellectual distance and taps into our most basic structures of sense-making. Merleau-Ponty (2013) writes that: “What brings about the hallucination and the myth is […] *not abolished but repressed by everyday perception* or by objective thought […]” (p. 304, italics added). This supports the idea that the mystical experience suspends the ordinary filters of conceptual mediation and reveal a more primordial layer of being - something which is always already present, but which we do not inhabit fully in ordinary consciousness. In this sense, the noetic quality is not the result of epistemic justification, but rather a result of a shift in the structure of perception itself. Mystical insight, then, might be seen not as irrational, but as an experience of intrinsic meaningfulness normally obscured by our habitual, instrumental modes of relating to the world. Again, noetic quality can be contrasted as the opposite of the affectively meaningless world of the depressed subject.

### Transcendence of Time

Finally, the last core mystical feature this analysis will address is the distortion of time. Merleau-Ponty (2013) argues that temporal perception is not the objective registering of any one single moment in a linear chain of temporal instances. He writes that “I do not pass through a series of nows whose images I would preserve and that, placed end to end, would form a line. For every moment that arrives, the previous moment suffers a modification” (p. 439). Hence, perception is temporally structured in a dynamic triadic framework (Howell, 2015). It consists firstly of retention, namely the past which is carried into the present. Secondly of primal impression which is the immediate perception of stimuli in a given moment. Lastly, of protention, our anticipation of what we will perceive next. This triad informs perception at any given moment, and its three constituents are inseparable in lived experience. Howell (2015) proposes the example of a melody, in the case of which a note is only meaningful in relation to the notes which have preceded it, and the anticipation of the notes yet to come.

One might argue that in the mystical experience, the structure of the phenomenal field shifts in such a way as to leave primal impression significantly more salient than retention and protention, the absence of expectation towards the future leaving an impression of time as suspended or annulled. Thus, this facilitates a radical shift in the structure of lived time in spite of objective time (i.e. what can be measured with a clock) remaining the same. It is worth noting that such alteration in lived time is not unique to mystical experiences, but also appears in everyday consciousness, such as in the instance of ‘flow states’ - deep immersion in a task, resulting in an experience of perceived temporal suspension as well as meaningfulness, and positive mood states (Csikszentmihalyi & Nakamura, 2010).

Drawing on Merleau-Ponty, this section suggests that mystical experiences grant phenomenal access to pre-reflective, embodied structures of meaning-making, thereby illuminating key phenomenal features of the mystical state such as ego-dissolution, ineffability, noetic quality, and distortion of time. This supports the notion of the mystical experience as structurally opposite of depression, hence possessing the potential to relieve depressive symptoms. Due to space constraints, this paper has not addressed the phenomenology of subjective time in depression. However, empirical studies indicate that individuals with depression often perceive time as passing unusually slowly (Thönes & Oberfeld, 2015), which contrasts markedly with the sense of timelessness characteristic of mystical states. In what follows, the discussion will examine a critical objection to this conception.

## Critical Reflections: Meaning Beyond Veridicality

One critical point one might raise in response to the above claim is this: If the psychedelic-induced mystical experience restores the intentional arc and thus facilitates perceptual engagement with the world, how come it may result in experiences which appear to be non-veridical? E.g. when a volunteer in the experiment at Johns Hopkins university reports from a mystical experience that “I was in the void” and that “time and space did not exist there” and despite having been lying on a sofa reports that “I was on my knees, hands clasped in front of me and I bowed to this force” (Barrett & Griffiths, 2018, p. 4). If in the psychedelic experience we are being brought into contact with our lived world, how come we may experience things that appear to be simply untrue of our physical environment? Merleau-Ponty (2013) writes that:

“What protects the healthy man against delirium or hallucination is not his reason […], but rather the structure of his space: objects remain in front of him, they keep their distance and […] they only touch him with respect. What brings about the hallucination and the myth is the contraction of lived space, the rooting of things in our body, the overwhelming proximity of the object, the solidarity between man and the world, which is *not abolished but repressed by everyday perception* or by objective thought […]” (p. 304, italics added).

This response to the objection is supported by the quote, namely that the apparent paradox dissolves when we consider Merleau-Ponty’s perceptual framework more closely. This response argues that psychedelic mystical experiences do not distort or mislead perception in a radical sense, but rather reconfigure the intentional arc and alter the salience of inherent features of perception. That is, features which are always present but repressed by everyday perception. This reframing matters for the paper’s central thesis: if psychedelic experience discloses rather than distorts, then even its most apparently hallucinatory episodes remain instances of the same recalibration of the intentional arc examined in Sects. "Psychedelic Experience as Phenomenological Restoration" and "Returning to Mystical Experience", rather than a separate, merely subjective phenomenon requiring independent justification.

Importantly, perception to Merleau-Ponty is crucially not a passive reflection of objective reality but an active, embodied opening to a horizon of meaning shaped by our existential orientation. The “veridical” in this sense is not about matching a world of fixed objects but about being affectively and practically attuned to the world as meaningful. Rather than being empirically false, the hallucinated image may serve as a metaphorical or affectively charged re-engagement with the world. For instance, seeing one’s own reverence at the mystery of existence as an experience of kneeling is not perceptual error but may instead be conceptualised as a bodily-symbolic integration of feeling, memory, and meaning. Even physical gestures can pass “[…] from their literal to their figurative sense, [bringing] forth a new core of signification through them” (Merleau-Ponty, 2013, pp. 147–148). The meaning we perceive in the world need not be limited to simple, concrete associations (e.g., “mice = dangerous”), but may take on rich symbolic dimensions. Such symbolic engagement is clearly a significant way in which meaning and emotional valence are experienced and communicated both in everyday life, in art, and in altered states of consciousness (Carter & Fuller, 2015; Balter, 2009).

Hence, the objection is misguided given that perception is never entirely veridical; however, a psychedelic experience may indeed be an instance of engagement with the world where certain elements (such as connectedness, meaning-making, etc.) appear far more salient than usual at the expense of other elements (such as the genuine presence of physical objects, my impending tax return, etc.). Merleau-Ponty (2013) makes the same point when he writes about hallucinations that: “[…] this fiction can only count as reality because reality itself is reached for the normal subject in an analogous operation” (p. 358, italics in original).

This is not to say that a deep mystical state would not be pathological or problematic if it were a chronic condition, since it would then disrupt the individual’s ability to carry out a number of projects (such as filing a tax return). However, given the transient nature of the acute effects, this reorientation towards a different kind of perception is not in itself problematic. In short, when the volunteer participant perceives himself as kneeling in a void suffused with Love, he is not necessarily misperceiving the world. Rather, he is engaging with a different pre-reflective mode of attunement, exactly the mode of attunement which is pathologically deficient in the depressed state.

In this section, this analysis has responded to a critical objection, emphasising that the seemingly non-veridical nature of psychedelic hallucination may be understood as a shift in perceptual salience, drawing experience towards features which are always present in perception, only normally more subtly so. The discussion will proceed to discuss the implications of this analysis for psychology.

## Discussion

This paper has argued that psychedelic therapy functions not merely by correcting chemical imbalances or implanting comforting beliefs, but by facilitating a re-experiencing of the world. Crucially, this approach does not reject scientific or neurobiological accounts but complements them by attending to what those accounts often overlook: the lived world of the patient. To better understand not just *that* psychedelic therapy works but how it works for the subject, further research is required which can capture the structure of lived experience before, during, and after treatment. In this context, qualitative methods become indispensable. Researchers may employ in-depth interviews or interpretative phenomenological analysis to systematically explore first-person accounts (Eatough & Smith, 2017). Especially valuable would be longitudinal studies investigating how patients integrate altered experiential perspectives into daily life over time. Such studies may help identify which integration practices are most effective for sustaining long-term therapeutic gains (Bathje et al., 2022). Further, while clinical findings are promising, it is important to address risks. Could such profound experiential shifts carry risks of misattunement or destabilisation? Might certain populations be more vulnerable to adverse outcomes? Future empirical studies must investigate whether such groups exist and, if so, whether risks can be mitigated through preparatory screening or tailored therapeutic protocols. This concern also has a phenomenological dimension and is not purely an empirical matter: Sect. "Critical Reflections: Meaning Beyond Veridicality" argued that a transient reweighting of perceptual salience is therapeutic, but that the same shift, were it to become chronic, would itself constitute a pathological disruption of the intentional arc rather than its restoration. A phenomenological framework may therefore help specify what a concerning outcome would consist in, and not only whether one has occurred.

As for psychedelic therapy protocols, many already reflect phenomenological insights. Most contemporary frameworks emphasise the importance of “set and setting” - a recognition that both internal factors (such as expectations, psychological history, and personality traits) and external conditions (such as the physical environment and therapeutic support) play a critical role in shaping the psychedelic experience. This principle is widely considered foundational to the safe and effective use of psychedelics in clinical context (Hartogsohn, 2017). Moreover, a phenomenologically-inspired approach would encourage not simply measuring symptom reduction, but aiming to ask the right questions in order to understand how the patient’s psychedelic experience has reshaped their structures of perception and meaning-making.

While the phenomenological framework has here been applied exclusively to depression, it is likely more broadly applicable to treatment of conditions such as anxiety and substance abuse (Feulner et al., 2023; DiVito & Leger, 2020). Due to space constraints, this could not be explored within this paper, but represents an interesting direction for future research.

## Conclusion

This paper set out to examine how Merleau-Ponty’s phenomenology can illuminate the role of psychedelic-induced mystical experiences in mediating therapeutic effect for depression. Central to this project was the rejection of Cartesian dualism, still latent in dominant psychiatric paradigms, in favour of a model of subjectivity as fundamentally embodied and intertwined with the world. This move directly answers the two objections with which the paper began. If therapeutic benefit is mediated by a change in the fundamental structure of experience, rather than by a discrete neurochemical event or by the endorsement of a comforting belief, then mystical experience is neither a merely epiphenomenal correlate of neuroplasticity nor a vehicle for metaphysical consolation, but the mechanism of change itself.

Drawing on the concepts of the body-subject and the intentional arc, this paper has argued that depression constitutes a breakdown in pre-reflective attunement to the world - a collapse in the very structures that render experience affectively and practically meaningful. In contrast, psychedelic-induced mystical experiences appear to restore this attunement by recalibrating the intentional arc, allowing the world to be re-experienced as affectively and perceptually salient. This reorientation is not merely cognitive, but entails a pre-reflective restructuring of how the subject perceives and inhabits the world.

Crucially, Merleau-Ponty’s phenomenology offers an account of core phenomenal features of mystical experience which stand in stark contrast to those of depression. Ego dissolution can be understood as a temporary suspension of habitual ego-boundaries and a widening of the intentional arc to let the interconnected with the perceptual world appear especially salient. The ineffability of the experience reflects its grounding in pre-reflective, embodied perception, which resists full conceptual articulation. The noetic quality can be explained as a heightened salience of embodied meaning-making, unmediated by habitual cognitive filters. Finally, the altered temporal experience often reported during mystical states can be illuminated through Merleau-Ponty’s model of temporality as a dynamic experiential structure. Thus, Merleau-Ponty’s framework not only clarifies the therapeutic mechanism of psychedelic-induced mystical experiences but also justifies this conception by accommodating the acute experiential dimensions of these.

## Data Availability

No datasets were generated or analysed during the current study.
